# ENTAIL: yEt aNoTher amyloid fIbrils cLassifier

**DOI:** 10.1186/s12859-022-05070-6

**Published:** 2022-12-01

**Authors:** Alessia Auriemma Citarella, Luigi Di Biasi, Fabiola De Marco, Genoveffa Tortora

**Affiliations:** Department of Computer Science, University of Salerno, Fisciano, Italy

**Keywords:** Protein classification, Amyloidoses, Fibrils machine learning

## Abstract

**Background:**

This research aims to increase our knowledge of amyloidoses. These disorders cause incorrect protein folding, affecting protein functionality (on structure). Fibrillar deposits are the basis of some wellknown diseases, such as Alzheimer, Creutzfeldt–Jakob diseases and type II diabetes. For many of these amyloid proteins, the relative precursors are known. Discovering new protein precursors involved in forming amyloid fibril deposits would improve understanding the pathological processes of amyloidoses.

**Results:**

A new classifier, called ENTAIL, was developed using over than 4000 molecular descriptors. ENTAIL was based on the Naive Bayes Classifier with Unbounded Support and Gaussian Kernel Type, with an accuracy on the test set of 81.80%, SN of 100%, SP of 63.63% and an MCC of 0.683 on a balanced dataset.

**Conclusions:**

The analysis carried out has demonstrated how, despite the various configurations of the tests, performances are superior in terms of performance on a balanced dataset.

## Background

In recent decades, protein sequencing techniques for determining the exact peptide sequence have resulted in the availability of a huge amount of data to be analyzed. In particular, in the biological area, it is critical to identify, classify, investigate [1, 2] and give known proteins a three-dimensional structure in understanding their function [3, 4].

Detecting anomalies in protein sequences and structures is especially crucial in circumstances when early detection is required to recognize illness onset symptoms. In this context, the role of protein misfolding is becoming increasingly important, where a change in a normal protein is the triggering event of a disease [5]. A mutation in the genes that encode the protein components of fibrillar aggregates can enhance protein misfolding and aggregation. Amyloidosis is one of those illnesses that begins with non-specific symptoms and represents a huge range of affections that are completely different from one another in terms of clinical, symptomatological, and, at times, prognostic factors.

Amyloidoses are a rare category of disorders caused by the extracellular deposition of amyloid, a fibrillar substance produced from multiple precursor proteins produced by the organism. These proteins self-assemble into highly ordered aberrant conformations and it deposited as tiny insoluble fibres in vital organs, causing damage. Amyloid is a form of protein that isn’t ordinarily found in the body, but it can be made up of a variety of proteins. Some types are transmitted from generation to generation. Others are caused by external factors, such as inflammatory disease or long-term dialysis.

There are more than 20 amyloidosis calls among the group of heterogeneous pathologies, including Creutzfeldt–Jakob, Alzheimer’s, Huntington’s, and prion diseases [6]. The type and severity of amyloidosis symptoms depend on the vital organs affected. Some forms of amyloidoses are systemic, while others are localized. The heart, kidneys, liver, spleen, neurological system, and digestive tract are just a few of the organs that can be damaged. So, some patients have minor symptoms, while others develop severe, potentially fatal pathologies [7]. The main obstacle in differentiating the various amyloidoses is the overlap of the clinical features across other pathologies. In this clinical outcome, cardiac problems are the leading cause of morbidity and mortality in amyloidosis. Amyloid fibril deposits in the heart cause restrictive cardiomyopathy with ventricular failure and, in advanced stages, hypotension and low cardiac output [8]. Systemic amyloidosis falls into four main categories, based on the protein composition of the amyloid deposits [9]:

- *Primary amyloidosis (AL)* Also known as amyloidosis from light chains, it is characterized by alterations in plasma cells that create aberrant antibody proteins in large quantities (light chains). The liver, the heart, the kidneys, the tongue, the intestine, the blood vessels, the skin, the nerves, and the spleen are the most common sites for primary amyloid deposits;
- *Secondary amyloidosis (AA)* It can occur as a result of infections or chronic inflammation, as well as after some types of tumours. The kidney is the most significantly affected organ in this scenario; however other organs may be damaged too;
- *Hereditary amyloidosis* It represents a collection of rare hereditary illnesses caused by mutations in certain blood proteins. Amyloid fibrils, which affect the kidneys, nerves, and heart, are caused by these altered proteins. The most common cause of recognized amyloidosis is altered transthyretin (ATTRm), a protein produced by the liver;
- *ATTRwt amyloidosis* It primarily affects the heart and is much more common in men than women. The cause of this pathology is linked to the abnormal conformation of the *wild type* (not altered) transthyretin.

Regardless of the nature of the amyloid protein that constitutes them, the amyloid fibrils seem to have all a similar structure. Therefore, X-ray diffraction techniques, Electron Microscopy (EM), and specific chemical staining are all methods that can be used to inspect it in vitro [10]. However, these methods have cost and time limitations, so computational methods for detecting and predicting fibrils have recently emerged in response to the large amount of protein data produced by sequencing.

In this study, our goal was to evaluate the behavior of the classifier purely on balanced amyloid fibril datasets, because the datasets used in the literature are almost always unbalanced. The performance on a balanced or an unbalanced dataset has an impact on the automatic learning of the different machine learning algorithms [11]. The research question is: What is the behavior of amyloid fibril classifiers in the presence of balanced datasets? We developed a classification system for amyloid fibrils trained on the balanced dataset to avoid learning bias during classification training. This system, designated as ENTAIL, employs 4125 descriptors that describe the structural and physicochemical properties of proteins. We explored two types of classifiers, Ensemble Classifier with GentleBoost and Naive Bayes Classifier, setting up different experiments in which cross validation and learning rate varied. After a training phase on the balanced dataset and a validation phase, we tested our model on observations that it has never seen before to evaluate its performance. The final method is based on the Naive Bayes Classifier and has been tested against existing predictors in the literature.

This paper is organized as follows. The details of the methods are presented in “Methods” section. Results are discussed in “Results and discussion” section. In “Conclusion” section, we summarized conclusions and future works.

### Related works

Tian et al.  [12] based on a support vector machine (SVM), proposed the Pafig (Prediction of the amyloid fibril-forming segment) method for identifying fibril-forming segments in proteins. Their model used 41 physicochemical properties from the Amino acid index database (AAindex). Pafig reached an overall accuracy of 81%, a specificity of 80%, a sensitivity of 82% and Matthews correlation coefficient of 0.63 [13].

Niu et al. [14] proposed RFAmyloid, a web server based on SVMProt 188-D feature representation, pse-in-one feature representation, and random forest classifier to identify amyloid proteins. The accuracy was 89.19% while the Matthews correlation coefficient, sensitivity, specificity, and F-measure were 0.739, 0.781, 0.927, and 0.891, respectively.

Li et al. constructed a machine learning-based prediction model called PredAmyl-multilayer perceptron (MLP). The authors combined seven feature extraction algorithms, SVMProt-188D and tripeptide composition (TPC). As a result, they achieved an accuracy of 91.59%, a specificity of 0.950, a sensibility of 0.836 and MCC of 0.798 [15].

Teng et al. [16] presented and created an accessible ReRF-Pred, a novel machine-learning based on a multi-feature encoding strategy for predicting amyloidogenic regions, using the pseudo amino acid composition (PseAAC) [17] and tripeptides composition (TPC) [18]. They obtained an accuracy, specificity, sensitivity and MCC of 80.10%, 83.1%, 73.4% and 0.552, respectively.

Burdukiewicz et al. [19] used n-grams and random forest in order to classify amyloidogenicity patterns based on the short portions known as hot spots that can trigger aggregation. Since the exact sequence of amino acids may not be necessary for amyloidogenicity, the authors tested 525284 reduced amino acid alphabets with varying letter lengths (from three to six) to determine which alphabet offered the highest cross-validation performance. Their predictor is called AmyloGram and reached 0.90 for Area under the ROC Curve (AUC) and 0.63 for MCC, a sensitivity of 86.5% and a specificity of 79%, with a length for the sequences between six and ten, on the pep424 data set.

Keresztes et al. [20] proposed a linear Support Vector Machine architecture (SVM) on the Waltz database of 1415 total hexapeptides [21]. These peptides are represented with two vectorial representations: in the first, the 20 amino acid names were simply translated into vectors; in the second, the authors used 553 properties derived from AAindex in order to describe the hexapeptides [12]. Their predictor is named *Budapest Amyloid Predictor* and they constructed 10 different SVMs with an accuracy between 73% and 86%. The AUC is 0.89.

## Methods

This section describes all the steps performed to obtain our classifier. We used MATLAB as the primary software. However, the common open-source packages that support AdaBoost and GentleBoost may be used.

### Datasets preparation

To build balanced and precise training and test sets, we used four datasets made available from the following projects: AmyLoad [22], WALTZ-DB 2.0 [21], AmyPro [23] and Pep424 [24].

*AmyLoad* is a website that collects amyloidogenic and non-amyloidogenic sequences with detailed information from the majority of known amyloidogenic datasets. It contains 1481 sequences (1037 non-amyloid peptides and 444 amyloid peptides); the sequences published on the AmyLoad website are listed in a table with columns identifying the name of the protein, the name of the fragment, the amino acid sequence, and the amyloidogenicity information. Furthermore, additional details are offered; however, they are presented separately for each fragment.

WALTZ-DB 2.0 is an open-access database with data on 1416 amyloid-forming hexapeptide sequences separated into nine source subsets: 515 amyloids and 901 non-amyloids were identified experimentally using electron microscopy, FTIR and Thioflavin-T experiments.

Pep424 is a dataset containing all available peptides annotated with experimental information: it contains 196 peptides, 158 hexapeptides, 70 peptides from human prion protein, human lysozyme, and $\beta$2-microglobulin. Therefore, Pep424 contains 424 peptides, 149 amyloid peptides, and 275 non-amyloid peptides.

AmyPro is an open-access database of validated amyloid precursor proteins. In particular, it is possible to download both sequence and amyloidogenic sequence regions from this database. We used the January 2022 version of AmyPro containing 168 entries (70 amyloid peptides) in this contribution.

**Fig. 1:**
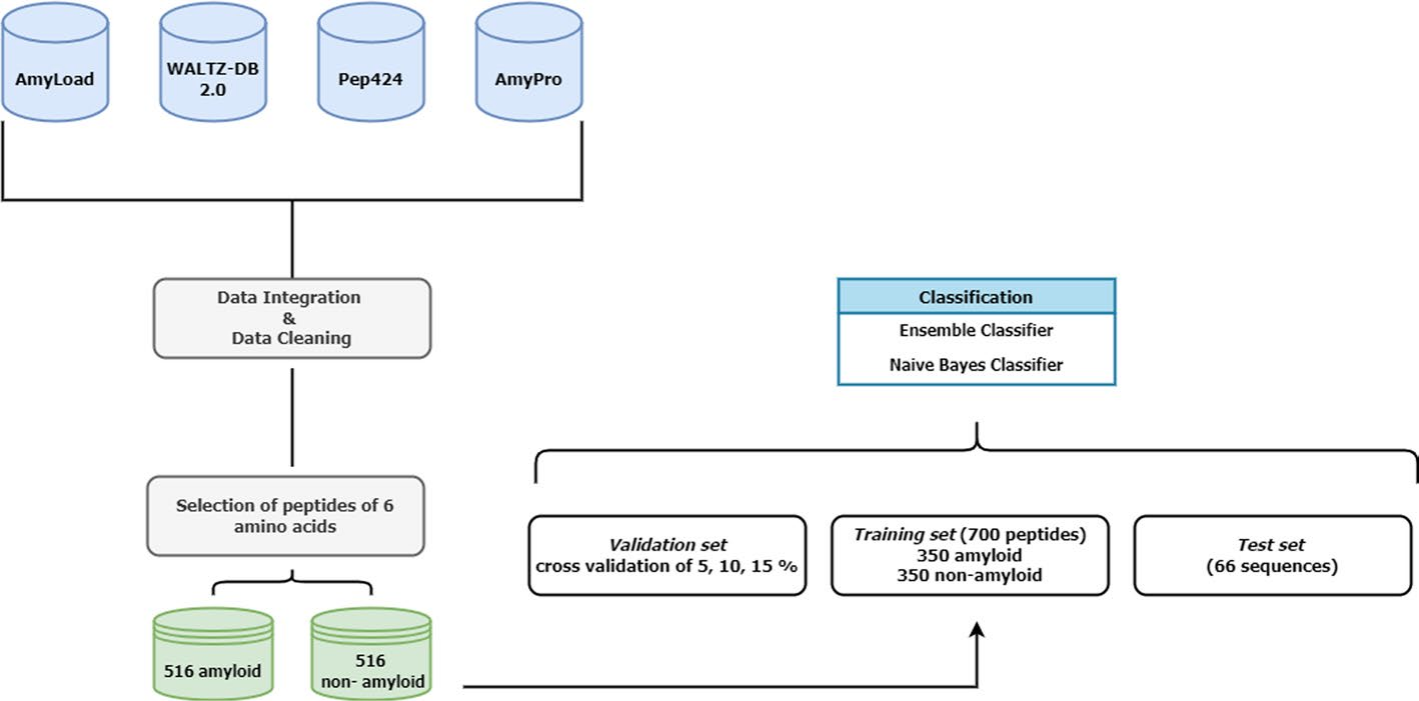
The composition of the aggregated dataset based on the length of the sequences

The experiment workflow is shown in Fig 1. Most sequences have a length of 6 amino acid residues for 1418 sequences, which corresponds to 74% of the entire dataset. Therefore, to provide the highest number of examples to the classifier Learner tool, we redefined the dataset based on the priority component represented by these sequences of length six. As a result, the final dataset was composed of 516 amyloid proteins and 900 non-amyloid proteins. Finally, we randomly eliminated 384 non-amyloid sequences. Please note that these eliminated sequences are reintroduced into the robustness classification step.

We divided the remaining dataset into three sub-datasets:

- *Training set* 700 proteins divided into 350 amyloids and 350 non-amyloids;
- *validation set* Built dynamically with cross validation of 5, 10 and 15% compared to 700;
- *Test set* 66 sequences never seen from the classifier.

Following that, we ran the Ensemble and Naive Bayes classifiers with different parameter settings, varying the values of cross validation, the max split, the number of learners and the learning rate.

### Feature extraction

In order to annotate the training sets to prepare a machine-learning training system to classify the amyloidogenic proteins, we have chosen the 4125 sequence descriptors recovered by iFeature, a Python toolkit for extracting sequences, structural, and physicochemical features from protein sequences [25]. In particular, we used the following descriptors categories: *QSOrder, SOCNumber, APAAC, PAAC, NMBroto, Geary, Moran, AAINDEX, ZSCALE, EGAAC, DDE, GAAC, GDPC, CTDC, CTDD.*

- *Quasi-sequence-order descriptors (QSOrder)* The distance matrix between the 20 amino acids is used to generate these descriptors;
- *Sequence order coupling number (SOCNumber)* It is a reflection of the effects of sequence order;
- *Amphiphilic pseudo amino acid composition (APAAC)* This feature is correlated to the amino acid composition and distribution in the hydrophobic and hydrophilic groups along the protein chain;
- *Pseudo amino acid composition (PAAC)* It considers the amino acid position in the peptide sequences and their frequencies;
- *Normalized Moreau–Broto (NMBroto), Geary autocorrelation and Moran autocorrelation* They represented molecular descriptors which encode both molecular structure and physicochemical properties, calculated in three different;
- *AAINDEX* AAindex is a database of numerical indices representing various physicochemical and biochemical properties of amino acids and pairs of amino acids [12];
- *ZSCALE* Z-scales are based on the physicochemical properties of the AAs, including NMR data and thin-layer chromatography (TLC) data.
- *Enhanced grouped amino acids content (EGAAC)* It is based on the grouped amino acids content (GAAC) feature, calculated with a window that moves from the N- to C-terminal of each sequence;
- *DDE*: The dipeptide deviation from expected mean feature vector [26];
- *Grouped amino acid composition (GAAC)* This descriptor divides the 20 amino acids into five groups based on their chemical–physical properties and computes the frequency of each group in a protein sequence. The five distinct groups are as follows: negative charge (D, E), aromatic group (F, Y, W), aliphatic group (A, G, I, L, M, V), and uncharged (C, N, P, Q, S, T);
- *GDPC* The Grouped Di-Peptide Composition encoding is another variation of the DPC descriptor;
- *CTDC* Taking the hydrophobicity attribute as an example, all amino acids are divided into three groups: polar, neutral and hydrophobic. The Composition descriptor consists of three values: the global compositions (percentage) of polar, neutral and hydrophobic residues of the protein;
- *CTDD* The Distribution descriptor consists of five values for each of the three groups (polar, neutral and hydrophobic) [27, 28], namely the corresponding fraction of the entire sequence, where the first residue of a given group is located, and where 25, 50, 75 and 100% of occurrences are contained.

All the datasets are available via GitHub^1^.

### Classifiers training

We performed the training and test step using the Classification Learner available in the Matlab *“Statistics and Machine Learning Toolbox”*. In addition, due to the high number of descriptors to explore, we extended the experimental setup with the *“Parallel Computing Toolbox”*. In Table 1, the configurations of the executed experiments are reported.

**Table 1:** Configuration of the experiments

ID experiment	Descriptors number	Validation type	Validation value	CC	Max split	Num learners	Learning rate	PCA
Exp 1	4125	Cross validation	5	EG	26	167	0.5915	No
Exp 2	4125	Cross validation	5	NB				No
Exp 3	4125	Cross validation	10	EG	22	Auto	0.0011	No
Exp 4	4125	Cross validation	10	EG	40	80	0.1	No
Exp 5	4125	Cross validation	10	NB				No
Exp 6	4125	Cross validation	15	EG	30	30	0.1	No
Exp 7	4125	Cross validation	15	EG	20	60	0.1	No
Exp 8	4125	Cross validation	15	NB				No

CC stays for Classifier Configuration. EG and NB mean Esemble;GentleBoost and Naive Bayers; Kernel Naive Bayes; Gaussian Kernel Type; Unbounded Support, respectively

The non-optimizable classifiers were trained via the standard Parallel Cluster feature. On the contrary, the optimizable classifiers were trained using one NVIDIA GTX 5000 to explore various possible configurations. We used “Bayesian optimization” as the optimizer and “Expected Improvement per second plus” as the acquisition function. The max number of iterations into the optimization case was 30.

The “Ensemble Classifier” and the “Bayes Naive” Classifier showed the best training and validation steps performances. After the preliminary training/validation step, we manually fine-tuned the classifier that exposed an accuracy greater than 0.81. As a result of the performance values (accuracy) given in the literature, this threshold (higher than 0.81) was chosen.

Thanks to the fine-tuning step, we were able to identify the following classifiers configurations capable of overcoming inaccuracy in the performance reported in [16]:

- Ensemble Classifier with GentleBoost as ensemble engine, Max Split 26, Learners 67, Learning rate 0.5915;
- Naive Bayes Classifier with Unbounded Support and Gaussian Kernel Type.

### Classifiers robustness

For the preliminary evaluation of the performances of the trained classifiers, we used the test set composed only of sequences that were not present in the training set. Also, to evaluate the robustness of the trained classifiers, we analyzed the performance of the classifiers considering the presence of intra-class dissimilarities and inter-class similarities. In particular, we took into consideration how simple modification in the dataset determines a change in the accuracy of the classifiers [29].

We followed the same approach used to evaluate the performance degradation of multiple Neural Network classifiers [30] on the melanoma detection problem, allowing the training set and the test set to slightly change by randomly rebuilding the datasets for each training iteration.

We reintroduced the randomly eliminated 384 non-amyloid sequences. Therefore, there is no control over what sequences were eliminated to build this step’s balanced training and validation sets.

In particular, we built 100 pairs of sets ($TV_i$ and $TS_i$): with $TV_i$, we identified the i-th TrainingSet, and with $TS_i$, we identified the i-th test set; Also, each $TV_i$ contained 350 amyloid sequences and 350 not-amyloid sequences while each $TS_i$ contained 33 amyloid sequences and 33 not-amyloid sequences; All the sequences in the dataset were of length six. Therefore, each $TV_i$ had a size of 700, and each $TS_i$ had 66. we checked the integrity of the experimental setup by avoiding duplicated pairs of ($TV_i$ and $TS_i$).

Finally, we recomputed all the descriptors and retrained the classifier 100 times using each dataset pair.

### Classifiers availability

In order to make the classifier available as a standalone tool, we compiled the GentleBoost version and the Bayes Naive version via Matlab Compiler MCC tools. All the source code and the compiled package are available via GitHub^2^.

### Performance measures

In order to evaluate model performance, we used *accuracy* (ACC), *sensitivity* (SN), *specificity* (SP), *Q*, and *Matthews correlation coefficient* (MCC) defined as follows:

1 $$\begin{aligned}&Accuracy\, (ACC) = \frac{TP+TN}{TN+FP+FN+TP} \end{aligned}$$

2 $$\begin{aligned}&Sensitivity\, (SN) = \frac{TP}{TP+FN} \end{aligned}$$

3 $$\begin{aligned}&Specificity \,(SP) = \frac{TN}{TN+FP} \end{aligned}$$

4 $$\begin{aligned}&Q = \frac{SN + SP}{2} \end{aligned}$$

5 $$\begin{aligned}&MCC = \frac{TP \times TN - FP \times FN}{{\sqrt{{(TP+FP)}{(TP+FN)}{(TN+FP)}{(TN+FN)}}}} \end{aligned}$$

where true positives, true negatives, false positives and false negatives are represented as TP, TN, FP and FN, respectively. The Matthews correlation coefficient represents the quality of a classification model, uniforming the classes of TP, TN, FP and FN of the confusion matrix. When the output classes have different sizes, MCC is more representative of the ACC because it assumes that all the classes in confusion matrix are adequately covered, even if one class is substantially under(or over) represented.

We calculated also the *Error Rate* and *Receiver Operating Characteristic curves* (ROCs) to ensure that *Area under the ROC Curve* (AUC) was greater than 0.5. The ROC curve depicts the performance of a classification model across all classification thresholds. The area under the complete ROC curve is measured by AUC, which is a composite measure of performance that takes into account all possible classification threshold.

The error rate is the percentage of incorrect predictions divided by the total number of instances; the formula is shown in the following formula:

6 $$\begin{aligned} Error Rate\, (RT) = \frac{FP+FN}{TN+FP+FN+TP} \end{aligned}$$

## Results and discussion

**Fig. 2:**
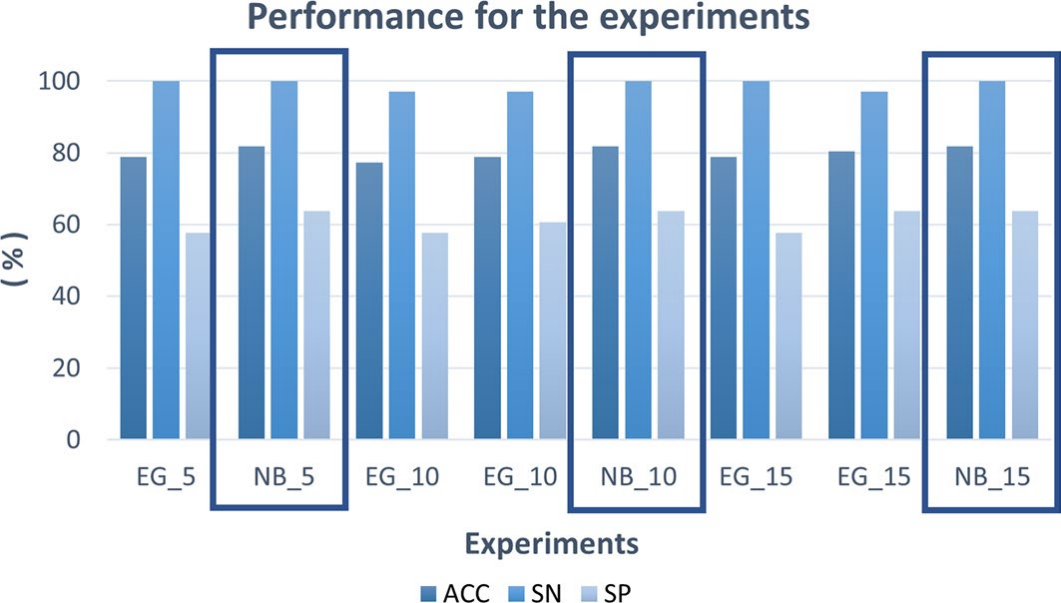
Comparison of the experiments and their configuration in the test phase

In Tables 2,  3, the TN, FP, TP and AUC values in validation and test phases are reported, respectively. In Tables 4, 5, we detailed the performance metrics of ACC, SN, SP, MCC and Q in validation and test phases. In Fig. 2, the performance of ACC, SN and SP are depicted for the different configuration of the experiments, as indicated in the Table 1 and 5, 10 and 15 indicate the values of cross validation. The boxes highlight the experiments with the best results.

In the validation phase, the best results, in terms of ACC are obtained in the Exp 3 and Exp 6, set with the parameters described in Table 1. Both classifiers are Ensemble Learning algorithms that use the GentleBoost method, varying the cross validation value. The value of ACC and Q for both the experiments are 88.10% and 88.14%,respectively. The SN is in the range of 86-86.86%, the SP is between 89.43-90.29% In the test phase, the most robust model is the Naive Bayes of experiments 2, 5, and 8 (Exp 2, Exp 5 and Exp 8), as the three parameters for cross validation vary. For the three experiments, the ACC is 81.80%, the SN, the SP. the MCC and Q values are 100%, 63.63%, 68.30% and 81.81%, respectively.

**Table 2:** TN, FP, FN, TP and AUC values in validation phase

ID experiment	TN	FP	FN	TP	AUC
Exp 1	309	41	46	304	0.94
Exp 2	261	89	41	309	0.82
Exp 3	316	34	49	301	0.95
Exp 4	308	42	52	298	0.94
Exp 5	263	87	41	309	0.82
Exp 6	313	37	46	304	0.94
Exp 7	310	40	46	304	0.94
Exp 8	262	88	38	312	0.82

**Table 3:** TN, FP, FN, TP and AUC values in test phase

ID experiment	TN	FP	FN	TP	AUC
Exp 1	19	14	0	33	0.88
Exp 2	21	12	0	33	0.83
Exp 3	19	14	1	32	0.88
Exp 4	20	13	1	32	0.89
Exp 5	21	12	0	33	0.83
Exp 6	19	14	0	33	0.89
Exp 7	21	12	1	32	0.91
Exp 8	21	12	0	33	0.83

**Table 4:** ACC, SN, SP, MCC and Q values in validation phase

ID experiment	ACC (%)	SN (%)	SP (%)	MCC (%)	Q (%)
Exp 1	87.60	86.86	88.29	75.20	87.57
Exp 2	81.40	88.29	74.57	63.50	81.43
Exp 3	**88.10**	**86.00**	**90.29**	**76.40**	**88.14**
Exp 4	86.60	85.14	88.00	73.20	86.57
Exp 5	81.70	88.29	75.14	64.00	81.71
Exp 6	**88.10**	**86.86**	**89.43**	**76.30**	**88.14**
Exp 7	87.70	86.86	88.57	75.40	87.71
Exp 8	82.00	89.14	74.86	64.70	82.00

Bold indicates higher values for performance

**Table 5:** ACC, SN, SP, MCC and Q values in test phase

ID experiment	ACC (%)	SN (%)	SP (%)	MCC (%)	Q (%)
Exp 1	78.80	100	57.57	63.60	78.78
Exp 2	**81.80**	**100**	**63.63**	**68.30**	**81.81**
Exp 3	77.30	96.96	57.57	59.30	77.27
Exp 4	78.80	96.96	60.60	61.80	78.78
Exp 5	**81.80**	**100**	**63.63**	**68.30**	**81.81**
Exp 6	78.80	100	57.57	63.60	78.78
Exp 7	80.30	96.96	63.63	64.30	80.30
Exp 8	**81.80**	**100**	**63.63**	**68.30**	**81.81**

Bold indicates higher values for performance

**Fig. 3:**
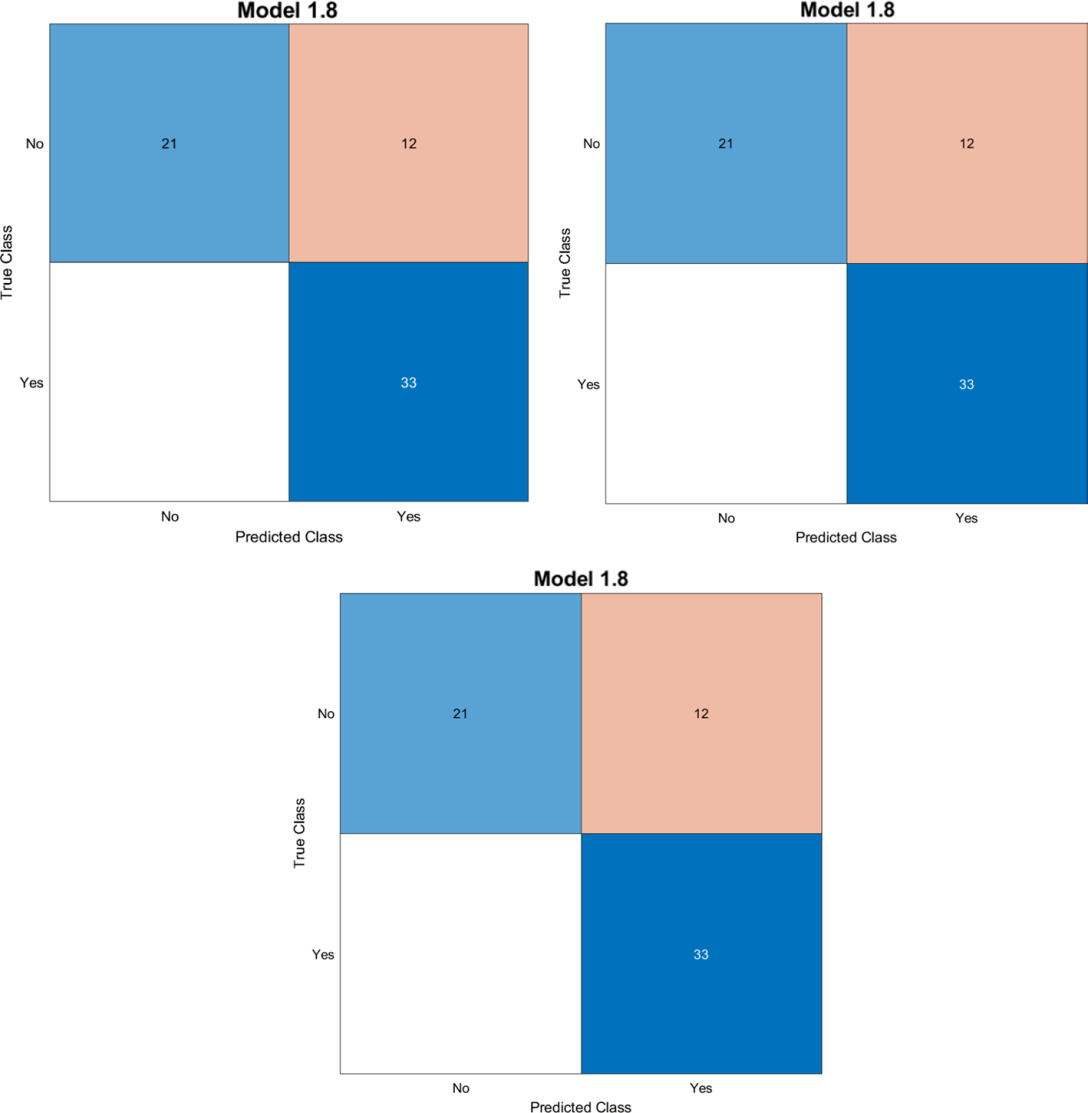
Confusion matrices for the best experiments. From left to right: experiment 2, experiment 5, and experiment 8

In Fig. 3, the confusion matrices of test phases are depicted for the three best performing classifiers. We can observe that all three Naive Bayes models properly classify the negative class more than the positive ones with an error of 18.2%, calculated with the formula (6).

**Fig. 4:**
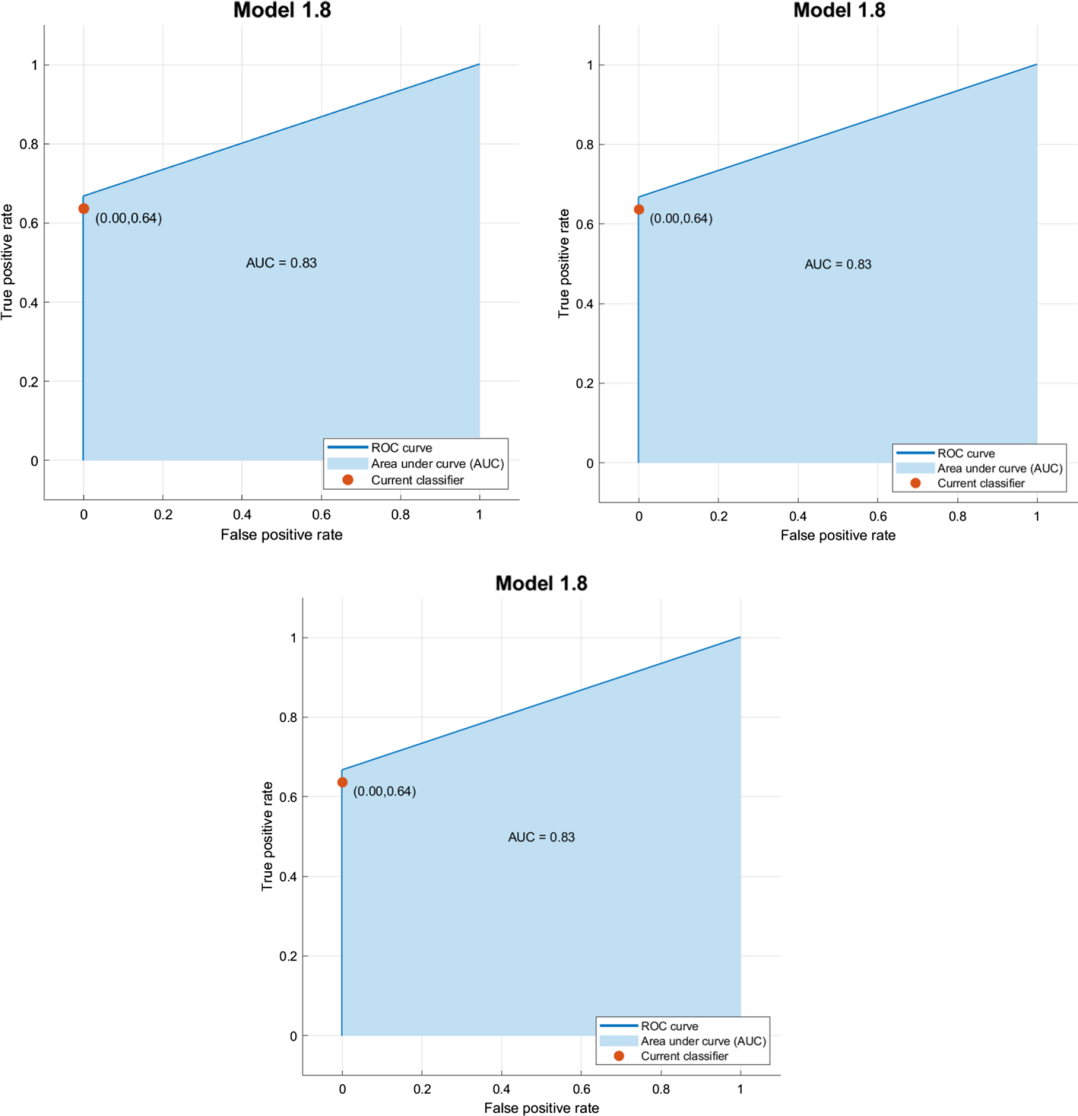
Roc Curves for the best experiments. From left to right: experiment 2, experiment 5, and experiment 8

In Fig. 4, we reported the related AUC calculated for the best classifiers (Exp 2, Exp 5 and Exp 8) in the test phase with 66 sequences never seen before by each classifier.

We calculated the robustness to determine how stable the ENTAIL classifier was while being tested on the new independent dataset. As a result, the “Bayes Naive” classifier resulted as the most robust across every value of the cross-validation fold (see Table 6). Finally, we evaluated all the classifiers with the PCA feature. We could not obtain a classifier with an accuracy greater than 0.81.

**Table 6:** Robustness in validation and test phase

Metrics	Validation robustness (%)	Test robustness (%)
Average of ACC	80.48	81.25
Average of SN	80.60	80.93
Average of SP	80.36	81.57

### Comparison with the literature

In Table 7, we summarized the comparison of ENTAIL with other existing methods in the literature.

Pafig is a predictor to identify amyloid hexapeptides based on SVM and which uses 41, 155, or 531 physicochemical properties for the description of sequences, selected with a standard genetic algorithm from AAindex [12]. The used dataset was Hexpepset, consisting of 1226 positive samples and 1226 negative samples. In this scenario, the value of MCC is better using only 41 physicochemical properties. The authors also tested the effect of different SVM kernel, founding that the best performance are reached with the use of Radial Basis Function (RBF) [13].

ReRF-Pred uses an unbalanced dataset as training set, with 511 amyloid and 903 non-amyloid samples. Using the PseAAC and TPC, the authors demonstrated and developed an accessible ReRF-Pred, a revolutionary machine-learning for predicting amyloidogenic regions. The authors specifically examined all possible tripeptides with binomial distribution, focusing on those with significantly different distributions between positive and negative samples [16]. PredAmyl-MLP and RFAmyloid train on a dataset consisting of 165 amyloid proteins and 382 non-amyloid sequences. PredAmyl-MLP extracts protein multi-feautures using a perceptron multilayer and two methods, SVMProt-188D and TPC. The first strategy is based on amino acid composition and physicochemical properties. The second represents protein tripeptide composition. These characteristics are integrated to encode each peptide sequence in the dataset, with a multi-feature of 8188 dimensions [15]. RFAmyloid is a random forest-based web server that uses SVMProt 188D, physicochemical characteristics, and the pse-in-one feature extraction approach for feature extraction, which consists of five groups of 22 feature extraction algorithms that yield all potential feature vectors for DNA, RNA, and protein sequences. The used dataset is Amy dataset, which consists of 165 amyloid proteins and 382 non-amyloid [31]. AmyloGram uses the n-gram extraction method and reduction of amino acid alphabet to determine which patterns in peptides are most important for their capacity to cause amyloid. Their method is trained on a final data set with 397 amyloid and 1033 non-amyloid sequences [19]. Budapest Amyloid Predictor takes as input data a hexapeptide and the the predictor returns a prediction for the sequence along with its 6 x 19 = 114 distance-1 neighbors. The models, based on 10 distinct SVMs, randomly chose 158 amyloid and 309 nonamyloid hexapeptides for the test set (corresponding to approximately 33%) out of the 1415 hexapeptides present in the Waltz database (514 amyloid and 901 nonamyloid peptides). Then, the remaining hexapeptides are used for training the linear SVMs models [20].

Our work is based on a balanced dataset, consisting of 350 positive and 350 negative samples. Compared to existing methods in the literature, ENTAIL classifies fibrils with a lower ACC and SP values but with a higher SN on the test set. The AUC is 0.83 and MCC is 0.683. From this comparison, despite the fact that our dataset is balanced, we can observe that the ACC values are not low and are similar to those of Pafig, the predictor that employs a balanced training dataset. The MCC value of ENTAIL is higher with respect to the MCC values of Pafig, ReRF-Pred and AmyloGram, trained on an unbalanced dataset. MCC reflects the quality of the input data. MCC can only get a high score if the classifier correctly predicted the majority of positive data instances and the majority of negative data instances. The existing methods use negatively imbalanced datasets [32]. Consequently, when the classifier succeeds at classifying negative cases, the SP increases to the disadvantage of SN. The increase in SN, with an error rate on FP of 18.2%, requires further analysis to understand the decrease in SP in the test set for ENTAIL peformance, although the presence of balanced positive and negative samples.

**Table 7:** Comparison with the literature

Methods	ACC (%)	SP (%)	SN (%)	MCC	AUC	Type of dataset
Pafig	81	80	82	0.63	–	balanced
PredAmyl-MLP	91.59	95.00	83.6	0.798	–	Unbalanced
ReRF-Pred	80.10	83.1	73.4	0.552	–	Unbalanced
RFAmyloid	89.19	92.7	78.10	0.739	–	Unbalanced
AmyloGram	–	79	86.5	0.63	0.90	Unbalanced
Budapest Amyloid Predictor	73–86	–	–	–	0.89	Unbalanced
ENTAIL (test set)	81.80	63.63	100	0.683	0.83	Balanced

## Conclusion

Current protein sequencing techniques operate in parallel, executing millions of reactions and collecting massive amounts of data. Many of these protein data obtained by sequencing must be appropriately classified and divided into protein families in order to understand their biological relevance in various organisms. Amyloidoses are a collection of rare and systemic diseases caused by deposits of aberrant proteins called amyloids in tissues and organs throughout the body. In this paper, we introduced ENTAIL, an amyloid fibril classification system based on the Naive Bayes Classifier with Unbounded Support and Gaussian Kernel Type, with an accuracy on the test set of 81.80%, SN of 100%, SP of 63.63% and an MCC of 0.683 on a balanced dataset of 350 amyloid and 350 non-amyloid samples. There are 4125 protein descriptors considered in the classification. Different classifier configurations were investigated in order to choose the best performing classifiers, the robustness of which was also estimated for both validation and testing. The results indicate that the ensemble algorithm using the GentleBoost approach produces the greatest results in the validation phase, while the Naive Bayers method provides the best results in the test phase with cross validation of 5 (Exp 2), 10 (Exp 5) and 15 (Exp 8). One potential direction for protein classification research is to improve classification system accuracy. In the future, we plan to explore amyloidogenic proteins further in order to discover the protein fragments involved for aberrant folding. To do this, we intend to provide new protein descriptors that will allow us to classify amyloidoses based on the different properties of insoluble amyloid fibrils.

## Data Availability

All the source code and the compiled package are available via GitHub. The dataset generated, all the source code and the compiled package used during the current study are available in the ENTAIL repository https://github.com/luigidibiasi/ENTAIL. Access to the repository is public.

## Abbreviations

AA: Secondary amyloidosis
AL: Primary amyloidosis
EM: Electron microscopy
MLP: Multilayer perceptron
SVM: Support vector machine

## Footnotes

1 https://github.com/luigidibiasi/ENTAIL/tree/main/classificator_inputs

2 https://github.com/luigidibiasi/entail/tree/main/Models/ENTAIL_3b
